# Social reward improves the voluntary control over localized brain activity in fMRI-based neurofeedback training

**DOI:** 10.3389/fnbeh.2015.00136

**Published:** 2015-06-03

**Authors:** Krystyna A. Mathiak, Eliza M. Alawi, Yury Koush, Miriam Dyck, Julia S. Cordes, Tilman J. Gaber, Florian D. Zepf, Nicola Palomero-Gallagher, Pegah Sarkheil, Susanne Bergert, Mikhail Zvyagintsev, Klaus Mathiak

**Affiliations:** ^1^Department of Psychiatry, Psychotherapy and Psychosomatics, Behavioral Psychobiology, RWTH Aachen UniversityAachen, Germany; ^2^Translational Brain Medicine, Jülich-Aachen Research AllianceJülich, Aachen, Germany; ^3^Department of Child and Adolescent Psychiatry, Psychotherapy and Psychosomatics, RWTH Aachen UniversityAachen, Germany; ^4^Department of Radiology and Medical Informatics, University of GenevaGeneva, Switzerland; ^5^Institute of Bioengineering, Ecole Polytechnique Fédérale de LausanneLausanne, Switzerland; ^6^Department of Child and Adolescent Psychiatry, School of Psychiatry and Clinical Neurosciences and School of Paediatrics and Child Health, Faculty of Medicine, Dentistry and Health Sciences, The University of Western Australia (M561)Perth, WA, Australia; ^7^Specialised Child and Adolescent Mental Health Services, Department of Health in Western AustraliaPerth, WA, Australia; ^8^Research Centre Jülich, Institute of Neuroscience and Medicine (INM-1)Jülich, Germany

**Keywords:** neurofeedback, real-time fMRI, social communication, reward, smile, avatar, Simon task, cognitive interference

## Abstract

Neurofeedback (NF) based on real-time functional magnetic resonance imaging (rt-fMRI) allows voluntary regulation of the activity in a selected brain region. For the training of this regulation, a well-designed feedback system is required. Social reward may serve as an effective incentive in NF paradigms, but its efficiency has not yet been tested. Therefore, we developed a social reward NF paradigm and assessed it in comparison with a typical visual NF paradigm (moving bar). We trained twenty-four healthy participants, on three consecutive days, to control activation in dorsal anterior cingulate cortex (ACC) with fMRI-based NF. In the social feedback group, an avatar gradually smiled when ACC activity increased, whereas in the standard feedback group, a moving bar indicated the activation level. In order to assess a transfer of the NF training both groups were asked to up-regulate their brain activity without receiving feedback immediately before and after the NF training (pre- and post-test). Finally, the effect of the acquired NF training on ACC function was evaluated in a cognitive interference task (Simon task) during the pre- and post-test. Social reward led to stronger activity in the ACC and reward-related areas during the NF training when compared to standard feedback. After the training, both groups were able to regulate ACC without receiving feedback, with a trend for stronger responses in the social feedback group. Moreover, despite a lack of behavioral differences, significant higher ACC activations emerged in the cognitive interference task, reflecting a stronger generalization of the NF training on cognitive interference processing after social feedback. Social reward can increase self-regulation in fMRI-based NF and strengthen its effects on neural processing in related tasks, such as cognitive interference. A particular advantage of social feedback is that a direct external reward is provided as in natural social interactions, opening perspectives for implicit learning paradigms.

## Introduction

People constantly control their brain activity by engaging in voluntary actions that are linked to activation of specific brain regions (deCharms et al., 2005; deCharms, 2007). This does not always work well: to excel in difficult skills, to suppress unwanted emotions or to override automatic actions requires a long and difficult learning process or even sometimes fails altogether. Although we do control the brain activity indirectly via our actions, typically we cannot exert direct control over specific brain regions. Brain imaging techniques, such as functional magnetic resonance imaging (fMRI), help to understand the link between the physiological processes taking place within the brain and our subjective awareness (deCharms, 2007, 2008). Neurofeedback (NF) based on real-time fMRI (rt-fMRI) takes us even a step further: Subjects can voluntarily change the activity in a selected brain region and directly see the effect of the evoked brain activation. Although this particular method is still relatively new and subject to certain limitations, its potential implications are vast.

NF based on electroencephalography (EEG) is well-established for the treatment of attention-deficit/hyperactivity disorder (Monastra, 2005) and epilepsy (Sterman and Egner, 2006) for over 4 decades now. fMRI-based NF, enabling the regulation of a precisely selected brain region, became available much later with the development of brain-computer interfaces (BCIs) based on rt-fMRI (for a review, see Weiskopf et al., 2004b, 2007). rt-fMRI NF proved to have a good anatomical resolution and to elicit behavioral changes (for a review, see Birbaumer et al., 2009). However, its use as a clinical method is still limited. One of the limitations is that only about two thirds of the people subjected to this particular method succeed in controlling computerized devices with brain signals, while the remaining one third fails to do so (Friedrich et al., 2014). A number of factors determine how well this control can be achieved, including the training protocol, instructions, tasks, mode of feedback as well as psychological traits such as motivation and expected reward, mood, locus of control, and empathy. Indeed, according to Goebel et al. (2004), the social component of the study paradigm, that is the willingness to compete and win against a real opponent, may lead to very fast and effective learning. In accordance with these observations, deCharms (2008) proposed to develop new types of task paradigms for rt-fMRI NF, where participants would be trained without engaging in a deliberate cognitive process.

In a standard fMRI NF paradigm, participants are presented with a visual display of a color bar moving up-and-down or a fluctuating thermometer that reflect brain activity in a region of interest (ROI). Their task is to raise the level on such a bar display by regulating the brain activity in the selected ROI. It is proposed that successful learning follows the principles of operant conditioning, involving a reward when the required threshold is achieved (McCarthy-Jones, 2012).

In EEG-based NF, the reward is often explicit, involving appointing points or making a game character move on the screen (Egner and Sterman, 2006) or make a Lego robot move forward (Mirković et al., 2013). In published rt-fMRI NF studies, the reward is often less direct such as the subject's own satisfaction with successful control of the bar display (or receiving social reward from an experimenter at the end of the task). deCharms et al. (2005) used task-related feedback stimulus, namely images of a fire changing its size to reflect a successful regulation of a pain-related area (rACC). Sokunbi et al. (2014) extended this particular approach, introducing feedback-guided self-regulation based on changing size of appetitive food pictures to regulate brain circuits related to hunger and food craving. They argued that the stimuli mimic avoidance behaviors during successful down-regulation and approach behaviors during unsuccessful down-regulation, increasing the face validity of the used training. Two remarkable studies applied explicit rewards in fMRI NF: Bray et al. (2007) offered monetary rewards when subjects successfully modified their motor cortex activity, and Goebel et al. (2004) added a social rivalry aspect in a so-called Brain-Pong game, where subjects played virtual ping-pong against each other, using their brain activity to control a racket (“brain-pong”). Although all the above attempts were successful, none was shown to be superior to standard feedback signals.

In daily life, we control our brain activity to change our facial expression, prosody, body posture, and other behavior based on subtle feedback signals that we receive from our partners in social interactions. Social reward, such as smile, can activate reward-related areas of the brain, similarly to other reward types, e.g., money (Izuma et al., 2008). We demonstrated in a pilot study that social reward can directly reinforce localized brain activity (Mathiak et al., 2010). In contrast to monetary reward (Bray et al., 2007), social reward can even be provided in real-time, i.e., by displaying positive facial expressions. Similar to the Brain-Pong setup (Goebel et al., 2004), where the inclusion of motivating (but not directly rewarding) social competition improved performance, social reward in the present approach may improve NF training by enhancing the motivation. Here, we investigated the impact of the feedback mode—smiling avatar face vs. standard bar display—on regulation performance during fMRI NF of the anterior cingulate cortex (ACC).

The ACC has been the focus of many studies due to its key role in regulating emotions, goal-directed behaviors, attentional processes, response selection, motor functions (Bush et al., 2000; Carter and van Veen, 2007), and above all in conflict monitoring and error perception (Botvinick et al., 2004; Kerns et al., 2004). The ACC can be successfully controlled using fMRI-based NF with standard paradigms (Weiskopf et al., 2003; deCharms et al., 2005; Emmert et al., 2014; Rance et al., 2014). However, in contrast to some visual and motor areas, no evident strategy emerged that yields activity increases without feedback mechanisms, so that the ACC is a suitable ROI to study learning in rt-fMRI NF. The ACC is reliably activated in both the color-word Stroop and the Simon tasks, which are both based on introducing interfering task-irrelevant stimuli (Peterson et al., 2002). In the Simon task, reactions to a target stimulus are slowed when the location of the target and the response side do not correspond, even though this is task-irrelevant. Both, the Stroop and the Simon task involve the ACC. However, in a direct comparison the Simon task led to significantly stronger ACC activations (Liu et al., 2004). Thus, we applied the Simon task to test for altered activation in the ACC region after NF training (generalization).

Twenty-four healthy participants were randomly allocated to one of two groups: a social and a standard feedback group. As for social reward, an avatar started gradually smiling when ACC activity increased. A bar display indicated the activation levels in the standard feedback group. To control for non-specific effects of the NF procedure, the subjects attempted to up-regulate their brain activity without receiving any feedback directly before (pre-test) and after the NF training (post-test). Further during the pre- and post-test, a cognitive interference task (Simon task) investigated change of ACC activity in a novel setting, also without feedback. The ability to voluntarily activate the ACC in an identical paradigm with no feedback served as measure for transfer of the NF training; the impact of the NF training on the ACC activity in the novel setting without feedback assessed generalization of the NF training (after Poppen et al., 1988; Simon and Gluck, 2013).

### Hypotheses

We expected successful NF training in both groups. In comparison with the control group, the social feedback group should demonstrate:

1. Stronger NF effect, i.e., higher activation in the ACC during NF sessions.
2. Higher activation of the reward system during NF sessions in response to the direct social reward.
3. Stronger transfer effects leading to higher activation during regulation without feedback; and
4. Enhanced generalization, i.e., a stronger effect on ACC activations during the post-test Simon task.

## Materials and methods

### Participants

Twenty-four right-handed subjects (13 females; age 25.62 ± 4.79) participated in the study. They were allocated based on the order of their inclusion in the study either to the social (12 subjects, 6 females, age 24.75 ± 2.80) or the standard feedback group (12 subjects, 7 females, χ^2^_(1)_ = 0.168, *p* > 0.682; age 26.5 ± 6.1, *t*_(22)_ = −0.891, *p* > 0.383). The alternating strategy did not exclude selection bias but minimized chronological bias (see Tamm and Hilgers, 2014). All participants were naïve to NF and reported the absence of any acute or history of major neurological or psychiatric disorder, any current use of psychoactive drugs as well as any contraindication for MRI. Written informed consent was obtained prior to participation. Afterwards, demographic information about age, gender, and education was collected. In addition, the participants were asked to complete The Positive and Negative Affect Schedule (PANAS; Watson et al., 1998) before and after NF training on each day. The study protocol was approved by the Ethics Committee of the Medical Faculty of the RWTH Aachen University, Germany, and the study was carried out in accordance with the Declaration of Helsinki.

### Experimental stimuli and task

#### Training

All subjects underwent standardized instructions for mental strategies to obtain voluntary control of localized brain activation (based on a written instruction set, see Supplementary Material). The instructions suggested to either recall positive emotional autobiographic memories, to imagine performing their hobby (like engaging in sportive or musical exercise), or to concentrate on a specific perception (like the temperature in one of their feet) in order to increase the activity in the ROI. The NF procedure was explained in detail, including the delay of the NF signal, and they were instructed to try each regulation strategy for at least 10 s. These instructions were delivered by the experimenter in a personal contact before the first measurement, and on the other days, participants received reminder of the task. Additionally, after each session the participants were asked which strategies they used in order to control their brain activity.

#### Design specification

All participants were trained to control their ACC activity by means of rt-fMRI NF on three separate days within 1 week. On each day, they performed three NF training sessions and two test sessions. We investigated neural correlates of three different conditions: (A) *NF*, i.e., up-regulation of the localized ACC activation with online feedback from the ROI signal (calculated over all nine NF sessions). The feedback signal here was either the bar or the avatar display; (B) *transfer*, i.e., up-regulation of the ACC ROI without feedback; and (C) *generalization* to a cognitive interference task, i.e., activity during the Simon task after NF.

#### Neurofeedback

**Figure 1:**
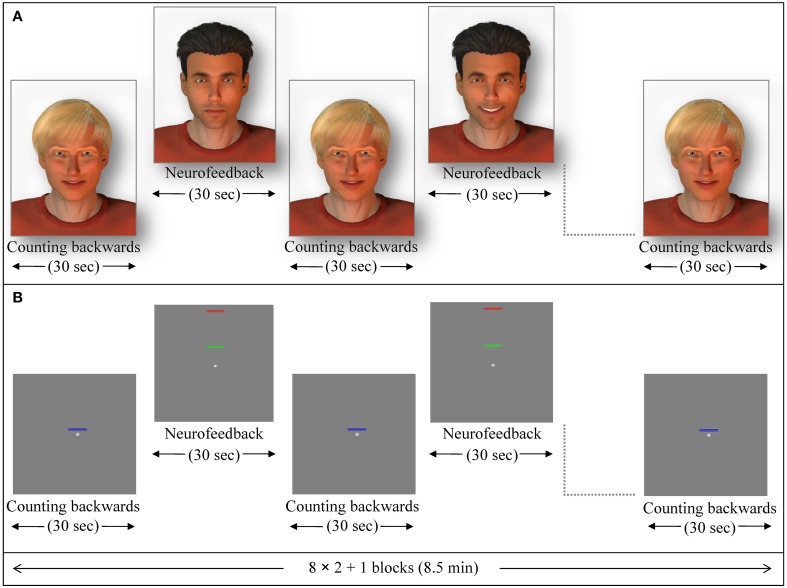
**Exemplary NF session**. Feedback blocks alternated with baseline blocks (backwards counting) of 30 s duration each. **(A)** In the social feedback condition, a dynamic avatar (here the one with dark hair) rewarded successful up-regulation with a smile while the other avatar (blond) indicated the baseline. From the completed datasets, 5 participants were studied in this configuration, whereas in the 7 other participants the blond one provided the feedback. **(B)** During the standard feedback condition, green bars moved toward the red one to indicate increase of activity while blue bars provided a cue to count backwards (baseline).

In the social feedback group, 12 participants received social feedback in which a male avatar (created with Poser Pro, Smith Micro Inc.), with either dark or fair hair (alternating and counterbalanced among subjects) provided a rewarding smile when subjects succeeded to increase ACC activity. The avatar became neutral when ACC activity decreased. The facial expression changed gradually within 100 frames. The second avatar was presented motionless and created a baseline (Figure 1A; see also Mathiak et al., 2010).

In the standard feedback group (control group), twelve further participants underwent the same ACC NF training. For this particular group, the change of ACC activity was indicated by either increase or decrease of a green moving bar. A blue motionless bar indicated the baseline condition in this group (Figure 1B; see also Gröne et al., 2014). Each NF session consisted of eight NF blocks and nine baseline blocks (30 s each; see exemplary session in Figure 1). The feedback was updated every repetition time (TR; 1 s). During baseline blocks, participants' instructions were to count backwards from 100.

#### Transfer

In order to test for the transfer of the NF training, subjects were instructed to regulate their localized brain activity without receiving feedback directly before (pre-test) and after (post-test) the NF sessions. The static stimuli from the NF training—the avatar or the green bar respectively—were presented in four blocks indicating to use a mental strategy to regulate ACC activity. As in the NF sessions, the baseline stimuli indicated to count-backwards (five blocks).

#### Generalization

**Figure 2:**
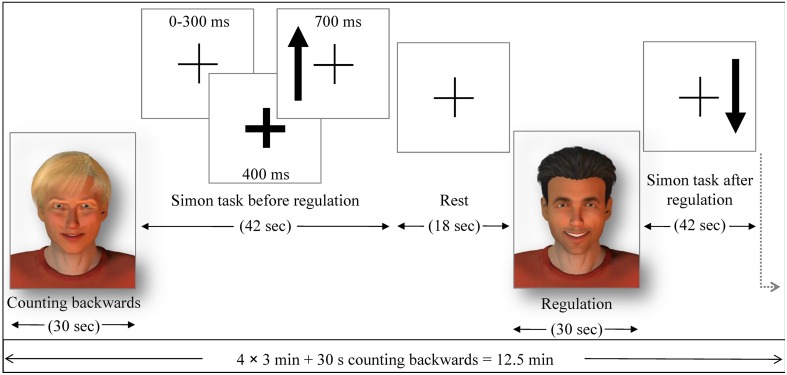
**The pre- and post-test runs**. Similarly as in the NF run, either the avatars or the bar display indicated the regulation without feedback (transfer) or baseline condition. Each regulation/baseline condition was followed by a Simon task (generalization), where participants had to indicate with left or right button press (within 700 ms response time) if the arrow presented on a screen pointed up or down The side where the arrow was presented constituted congruent and incongruent trials. The occurrence of an arrow was announced 400 ms in advance by a thickening of the continuously presented fixation cross. The average trial duration was 1250 ms (with 300 ms jitter), yielding 32 trials in 42 s and followed by a 18 s rest period, where subjects passively viewed the fixation cross.

In order to test for behavioral effects of the ACC NF and for a generalization effect of the training, a cognitive visuo-spatial interference task, an adapted version of a Simon task, was conducted in the pre- and post-test. A fixation cross was presented in the middle of the screen and accompanied by arrows pointing up or down on either the left or the right side of the cross (Figure 2). The participants responded with the button press (right or left) to the direction of the arrow (up or down). Thus, when the subject had to press the button on the opposite side of the arrow, a conflict occurred (incongruent trials). The Simon task was presented in eight blocks of 42 s each. The response buttons were counterbalanced between subjects and the events were presented in a pseudo-randomized order. Reaction time and accuracy of each trial were collected as behavioral measures during the Simon task. The stimulation for the transfer and generalization task was programmed with Presentation software (Version 16.3, www.neurobs.com).

### Data acquisition and analyses

fMRI scanning was conducted using a three Tesla whole body scanner (Magnetom TIM TRIO, Siemens, Erlangen, Germany). Echo planar imaging (EPI) covered 16 transverse slices parallel to the AC-PC line at a repetition time TR of 1 s (echo time TE = 28 ms; 64 × 64 matrix with 3 × 3 mm^2^ resolution; 3 mm slice thickness plus 0.75 mm gap). We obtained 520 volumes for each NF training run (about 8.5 min) and 760 volumes for each pre- and post-test (12.5 min). A custom made anatomical template of the ACC defined the ROI (Mathiak et al., 2010).

Online spatial preprocessing of the acquired brain volumes was conducted using a custom toolbox based on standard SPM procedures (Koush et al., 2012). In short, motion correction used spline interpolation with co-registration to the preselected template. The NF signal was extracted from each voxel in the ROI during the NF conditions, averaged for each volume and calculated as percentage of signal change relative to the preceding baseline block. Low frequency drifts were removed with an exponential moving average algorithm to improve the signal-to-noise ratio. A modified Kalman filter reduced outliers and high-frequency fluctuations. For feedback, the signal was rescaled in a fixed ratio such that about 1% signal change represented the full scale from neutral to maximally smiling face or from lowest bar position to the high target. Real-time analysis was performed on a separate PC using a custom Matlab toolbox for online fMRI preprocessing, analysis, and online feedback (for details on the online processing, see Koush et al., 2012).

Offline analysis of the imaging data comprised standard preprocessing and first level analysis in a block design. For the main effect, all runs and days were averaged since no specific time course of learning could be predicted. Group analysis was implemented as second-level two-sample *t*-test using the rather conservative family-wise error (FWE) correction for whole brain analysis and confirmative ROI analyses. In detail, the mapping analysis consisted of standard preprocessing steps with realignment, normalization, resampling with 2 mm isometric voxels, and smoothing (8-mm full-width at half-maximum Gaussian kernel) with SPM8 (FIL, http://www.fil.ion.ucl.ac.uk/spm/). The first 10 volumes of each run were excluded from the analyses to account for T1-saturation effects. For the NF runs, the regulation was modeled in a block design applying a generic hemodynamic response function. Transfer and generalization conditions were modeled in a block design as well. *T*-maps for contrasts of interest in the second-level group analyses were corrected for multiple comparisons across the volume using FWE correction and are shown at corrected threshold (*p* < 0.05). For data exploration, interaction of transfer and learning in the social reward condition are presented for a voxel-wise uncorrected threshold (*p* < 0.001). Threshold for cluster extend was always 15 voxels. Anatomical labeling was conducted in accordance with the Anatomy toolbox for SPM8 (Eickhoff et al., 2005).

In addition to the whole brain analyses, we conducted ROI analyses using small volume correction focusing on the ACC and on the reward system, respectively. Thereby we could specifically address the hypotheses 1–4 and ensure that signal changes encompassed the ACC or reward system ROI. The definition of the ACC was based on three-dimensional probability cytoarchitectonical maps, which offer a precise tool for the localization of brain functions as obtained from functional imaging studies (Amunts et al., 2007; Zilles and Amunts, 2010). The mask for the reward system comprised putamen and caudate nucleus as well as globus pallidus and was created using WFU PickAtlas toolbox for SPM8 Maldjian et al., 2003). Activation clusters were displayed at a threshold according to *p* < 0.05 FWE-corrected for the small volumes with cluster size bigger than 15 voxels.

For data exploration, we extracted average hemodynamic responses from ROIs for ACC and the reward system and—as baseline control—from bilateral parieto-occipital clusters (MarsBaR toolbox; Brett et al., 2002). Correlation between ACC regulation and reward responses were calculated. To study learning effects over the runs and sessions, the baseline-corrected ACC ROI signal entered into a repeated-measures ANOVA using linear predictors for *run* and *day* and the inter-subject variable *group*. All calculations were performed using Matlab 2010b (The Math Works, Natick, MA).

## Results

### Behavioral data

Social and the standard feedback group did not differ with respect to the demographic variables age [*t*_(22)_ = −0.891, *p* > 0.383] or education [*t*_(22)_ = −0.266, *p* > 0.792]. For the positive affect subscale of the PANAS, repeated-measures ANOVA revealed significant main effects of days [*F*_(2, 42)_ = 11.829, *p* < 0.0001; day 1: 27.2 ± 0.9, d2: 24.0 ± 1.3, d3: 23.7 ± 1.3] and session [before vs. after fMRI measurement; *F*_(1, 21)_ = 5.801, *p* < 0.025; before: 26.0 ± 1.0, after: 23.9 ± 1.3]. Neither group [*F*_(2, 42)_ = 1.709, *p* > 0.521] nor the interactions between group and days [*F*_(2, 42)_ = 1.709, *p* > 0.193] and session [*F*_(1, 21)_ = 0.329, *p* > 0.572] yielded a significant effect. The negative affect exhibited the same pattern [days: *F*_(2, 42)_ = 11.829, *p* < 0.0001, d1: 11.9.3, d2: 11.0 ± 0.2, d3: 10.7 ± 0.2; session: *F*_(1, 21)_ = 16.774, *p* < 0.025; before: 11.7 ± 0.3, after: 10.7 ± 0.2; group or interaction with group: all *p* > 0.09]. In summary, the random allocation yielded comparable groups and general blunting over time but no effect of the feedback strategy on the reported mood emerged.

Reaction times and accuracies of responses collected during the Simon task were assessed with ANOVAs for repeated measures. One participant was excluded from this analysis due to missing data (from the standard feedback group). Since the sphericity assumption was violated for days [Mauchley's test χ^2^_(2)_ = 15.88, *p* < 0.0001] and for the interaction of days with congruency [χ^2^_(2)_ = 6.2, *p* < 0.045], the Greenhouse-Geisser correction was applied. Days [*F*_(1.29, 27.13)_ = 15.065, *p* < 0.0001], session [pre- vs. post-test, *F*_(1, 21)_ = 11.731, *p* < 0.003] and the Simon effect [*F*_(1, 21)_ = 43.301, *p* < 0.0001] yielded significant effects on the reaction time. Subjects responded faster over the 3 days (day 1: 535.8 ± 12.4; day 2: 501.4 ± 11.2: day 3: 491.8 ± 14.0 ms) faster during post- than pre-tests (pre: 520.1 ± 13.2; post: 499.2 ± 10.7 ms), and faster during congruent than incongruent trials (congruent: 490.7 ± 10.8, incongruent: 528.6 ± 13.1 ms). Accuracy was only affected by congruency [*F*_(1, 21)_ = 26.318, *p* < 0.0001; congruent: 97.4 ± 0.8%, incongruent: 94.6 ± 0.8%]. In summary, a clear effect of stimulus congruency on performance in the Simon task was replicated and training speeded the responses, but no effect of the specific NF training on behavior emerged.

### Neurofeedback

**Figure 3:**
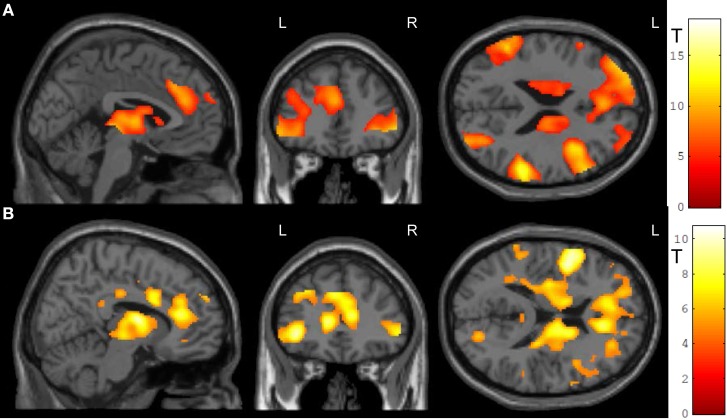
**NF training.(A)** Both modes of neurofeedback led to increased activity in ACC and in reward-related brain areas. **(B)** In the social feedback group, activity was higher in bilateral ACC and in the reward system as compared to the standard feedback group. Moreover, clusters in prefrontal, occipital, and temporal lobe emerged in this group comparison as well (see Table 1 for details). All maps are displayed at a threshold according to *p* < 0.05, FWE-corrected.

In the feedback runs, a distributed network was more active during NF as compared to the counting backward baseline (Figure 3A). In addition to the ACC, this network comprised bilateral lateral occipital complex, striatum, and dorso-lateral prefrontal cortex. In contrast, activation decreased in bilateral posterior insula, postcentral gyrus, and the posterior cingulum (Table 1A). Masking with the anatomically defined ACC and reward system confirmed the localization of this activation pattern to encompass the ACC (MNI = [−4, 28, 36], *t*_peak_ = 10.02, *p*_FWE_ < 0.0001) and the reward system with peaks in bilateral caudate nucleus (left: [−12, 6, 14], *t*_peak_ = 11.94, *p*_FWE_ < 0.0001; right: [14, 2, 18], *t*_peak_ = 13.13, *p*_FWE_ < 0.0001).

**Table 1:** **Activation clusters during NF training**.

**Region**	**Peak MNI coordinates**			**Cluster size**	***T*-value**	***p*-value (FWE)**
	**x**	**y**	**z**			
**(1A) Neurofeedback**						
Right inferior temporal gyrus	48	−58	0	4545	18.47	0.0001
Right inferior frontal gyrus pars orbitalis	42	26	2	19418	17.49	0.0001
Left superior temporal gyrus	−54	−50	28	3462	11.59	0.0001
Left middle frontal gyrus	−40	12	42	206	7.10	0.0001
ACC	−8	−20	34	41	6.19	0.0001
**(1B) Neurofeedback: social > standard feedback**						
Left inferior frontal gyrus	−48	4	10	24583	10.67	0.0001
Dorsal ACC	−2	−18	38	2721^*^	10.67	0.0001
Rostral ACC	10	34	16	2144^*^	8.46	0.0001
Right primary visual cortex	20	−76	12	178	6.40	0.0001
Right inferior parietal cortex	46	−54	42	530	7.82	0.0001
Left lingual gyrus	−18	−70	2	152	6.45	0.0001
Right primary visual cortex	20	−76	12	178	6.40	0.0001
Right rolandic operculum	−54	−18	22	186	6.34	0.0001
Left middle occipital gyrus	−18	−70	2	152	6.45	0.0001
Left inferior occipital gyrus	−46	−72	2	184	6.21	0.001
Left middle temporal gyrus	−58	−32	−4	49	5.38	0.002

* *The cluster sizes for the ACC were calculated in a mask based on three-dimensional probabilistic cytoarchitectonic maps*.

The group comparison revealed a higher effectiveness of the social NF over the standard feedback, as demonstrated by a significantly higher bilateral ACC activity (*t*_peak_ = 10.67, *p*_FWE_ < 0.0001; Figure 3B). Furthermore, an extended activation cluster emerged encompassing bilateral inferior frontal gyrus, the left occipital gyrus, and the left middle temporal gyrus (Table 1B). Anatomical ACC and reward system masks confirmed the localization of higher activation during social feedback in the ACC ([−10, 34, 10], *t*_peak_ = 9.00, *p*_FWE_ < 0.0001) and the reward system bilaterally with peaks in bilateral putamen (left: [−32, −10, 2], *t*_peak_ = 9.51, *p*_FWE_ < 0.0001; right: [36, 0, −4], *t*_peak_ = 9.06, *p*_FWE_ < 0.0001). Thus, hypotheses 1 and 2 were confirmed with higher ACC and reward system activity during social feedback. Notably, the average responses in the ACC ROI correlated with the one from the reward system [*r*_(24)_ = 0.535, *p* = 0.0071], suggesting a direct relationship of reward processing and learning success.

**Figure 4:**
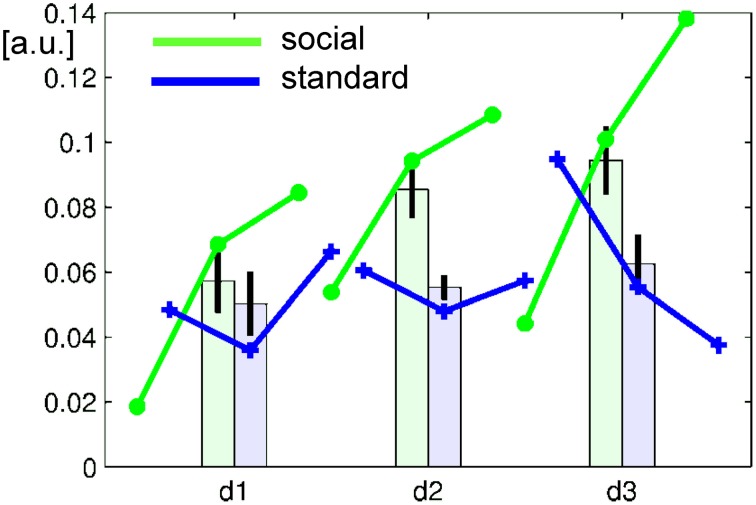
**Learning curve over runs (lines) and days (bars)**. In the baseline-corrected ACC ROI, the average signal increased in the social feedback group (green), but less so with standard feedback (blue). Repeated-measures ANOVA revealed a significant *group* × *days* interaction confirming a significantly stronger increase over days after feedback display with faces than with bars. Error bars represent the 95%-confidence interval for the repeated-measures estimator.

Learning of NF related regulatory control may be associated with increase of signal change over time. After baseline correction for the bilateral parieto-occipital junction clusters, average signal change in the ACC ROIs revealed a complex learning pattern influenced by the repetition over three runs on 3 days each (see Figure 4). Learning curves in NF may be complex and highly non-linear (Sarkheil et al., 2015), but frequently are approximated by linear curves. Therefore, repeated-measures ANOVA included runs and days as separate linear predictors and revealed a clear *days* × *group* interaction [*F*_(1, 23)_ = 8.239, *p* < 0.0089] but no main effect or other interaction [all *F*_(1, 23)_ < 1.8, *p* > 0.19, except a trend for *days, F*_(1, 23)_ = 3.022, *p* = 0.0961]. Further, the probability that individuals achieved control over the signal was estimated on their run-wise success rate and varied across subjects but not between the groups (mean ± SD: 69.4 ± 32.3%). In summary, the differential signal increase observed in the ACC seemed stronger in the social feedback group across runs as well as days, which was statistically confirmed for a stronger linear increase across days only.

### Transfer

**Figure 5:**
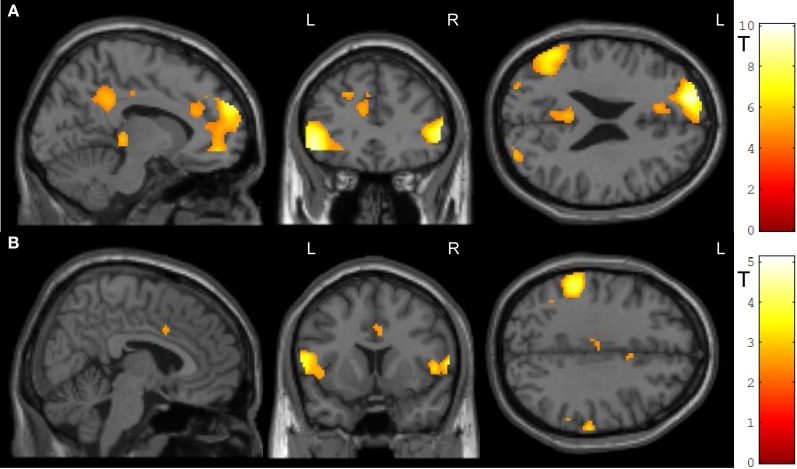
**Transfer. (A)** In both groups, ACC activity increased during transfer condition i.e., regulation without feedback (*p* < 0.05, FWE-corr.). **(B)** Social feedback led to higher transfer than standard feedback, although this activation did not survive the correction (*p* < 0.005, uncorr.). Further, the social learning group yielded higher activity in the left inferior frontal gyrus and the left inferior parietal cortex (see Table 2).

Transfer conditions revealed significantly higher ACC activity during the post-test regulation blocks without feedback compared to baseline blocks; in addition to ACC activity, distributed activation clusters emerged in bilateral inferior frontal gyrus and occipital gyrus, in the right middle occipital and middle temporal gyrus, left posterior cingulate cortex as well as thalamus (Figure 5A, Table 2A). ROI masks confirmed localization of activity in the ACC ([−10, 32, 24], *T*_peak_ = 5.23, *p*_FWE_ < 0.0001).

**Table 2:** **Activation clusters during transfer**.

**Region**	**Peak MNI coordinates**			**Cluster size**	***T*-value**	***p*-value (FWE)**
	**x**	**y**	**z**			
**(2A) Transfer**						
Left inferior frontal gyrus extending in ACC and others	−52	24	8	5138	10.02	0.0001
Right V5	36	−78	10	975	8.40	0.0001
Right inferior frontal gyrus pars triangularis	54	34	6	540	8.31	0.0001
Left middle occipital gyrus	−36	−80	14	3627	7.61	0.0001
Right middle temporal gyrus	46	−54	4	460	6.21	0.0001
Posterior cingulate cortex	−4	−40	34	995	6.06	0.0001
Left thalamus	−12	−30	0	164	5.64	0.0001
Right superior temporal gyrus	66	−40	18	19	4.66	0.018
**(2B) Transfer: social > standard feedback**						
Left inferior frontal gyrus pars opercularis	−50	2	12	1294	5.11	0.003
Left inferior parietal cortex	−54	−36	42	1001	4.57	0.026
Right inferior frontal gyrus pars opercularis	50	16	4	713	4.24	0.0001 (uc.)
Right inferior parietal cortex	66	−24	36	436	3.30	0.001 (uc.)
Right inferior parietal cortex	48	−42	46	78	2.97	0.002 (uc.)
Left middle cingulate cortex	−6	−22	40	100	2.96	0.002 (uc.)
Right Insula	42	−18	−6	23	2.75	0.003 (uc.)
Right middle frontal gyrus	30	42	20	28	2.60	0.005 (uc.)
ACC	6	10	34	25	2.58	0.005 (uc.)

uc.: uncorrected p-value

To test the prediction that transfer may differ between the two learning conditions, the interaction of transfer and learning groups was calculated. Indeed, during regulation blocks higher ACC activity was found in the social feedback group as compared to standard feedback (Figure 5B, Table 2B) but this interaction survived only an uncorrected threshold (*p* < 0.005) with an cluster-extend threshold of 15 voxels. Only peaks at the left inferior frontal gyrus and inferior parietal cortex survived the FWE-correction (Table 2B). The ROI analysis indicated higher regulation increase of the ACC in the social feedback group, but the peak did not survive the FWE-correction (MNI = [6, 10, 34], *T*_peak_ = 2.58, *p*_uncorr_ < 0.005). Lacking a higher activation in the social transfer condition after FWE-correction, we could not confirm Hypothesis 3.

### Generalization

**Figure 6:**
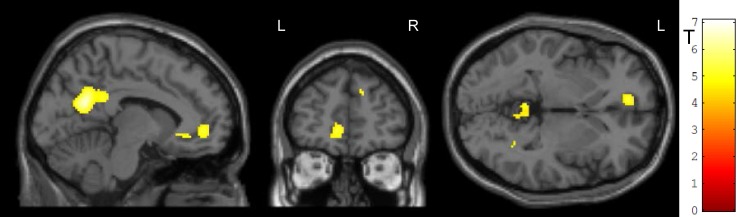
**Generalization**. The social feedback led to lesser ACC activity during interference processing in the Simon task (*p* < 0.05, FWE-corr.). The same comparison revealed a prominent cluster at the precuneus as well (see Table 3).

Generalization was tested as the effect of the transfer (regulation without feedback) on a subsequent block with the cognitive interference task, i.e., the group-by-task interaction during the Simon task. We found that ACC activation during cognitive interference processing was reduced after social reward compared to standard feedback. Higher ACC activity emerged in the non-social feedback group compared to social feedback group (*T*_peak_ = 5.34, *p*_FWE_ < 0.001; Table 3; Figure 6). ROI analysis confirmed the localization in the ACC (MNI = [−8, 34,−6], *T*_peak_ = 4.63, *p*_FWE_ < 0.001). Hypothesis 4 stated stronger effects on ACC activity during the generalization task after social NF training and this was corroborated by the data.

**Table 3:** **Group comparison of generalization (Simon task)**.

Simon task (transfer^*^group)	**Peak MNI coordinates**			**Clustersize**	***T*-value**	***p*-value (FWE)**
**Region**	**x**	**y**	**z**			
ACC	−12	34	−8	168	5.34	0.001
Precuneus	−4	−60	22	2225	7.06	0.0001
Left inferior parietal cortex	−34	−64	20	691	6.28	0.0001
Right middle temporal gyrus	48	−58	22	241	5.92	0.0001
Left middle frontal gyrus	−28	16	42	24	5.08	0.003
Right fusiform gyrus	34	−58	0	22	4.93	0.007

## Discussion

The present study investigated the effectiveness of social reward in rt-fMRI NF training of the ACC and compared it to a standard-type feedback in form of a moving bar. As predicted, social reward led to stronger ACC activity during NF training. After the training, both groups were able to regulate ACC activity without receiving feedback, with a trend for better performance in the social feedback group. Furthermore, during a cognitive interference task a significant difference for ACC activation emerged suggesting stronger generalization of the social feedback training on cognitive processing.

We extended previous studies using monetary reward (Bray et al., 2007) and created an innovative NF training based on a real-time social reward. In operant conditioning, a desired response is repeatedly paired with reward, resulting in increasing probability that the response occurs again. A conscious process is not necessary for the learning to take place. Although NF is believed to be based on principles of operant conditioning, no reward is delivered for a correct response in typical fMRI NF paradigms. The learning requires instead the explicit knowledge of the task in order to perform it correctly. Although changing the size of the color bar according to instruction can be satisfying as it signals success (the own satisfaction serves as a reward in this case), in a different context, e.g., during watching a movie with a color bar changing, it would not represent a rewarding value. Bray et al. (2007) made a first step in implementing an implicit feedback in a behavioral shaping paradigm; subject's responses were gradually changed by reinforcing small changes leading to a desired target behavior (Dinsmoor, 2004). The subjects did not need to have explicit knowledge of the task, but learned it gradually via receiving or missing a financial reward, depending on their performance. Although monetary reward constitutes a strong reinforcer, it is difficult to deliver in a real-time feedback in order to gradually shape the behavior.

Emotional expressions aim to communicate our experiences and to influence the behavior of others (Horstmann, 2003). Social reward offers therefore a more ecologically valid paradigm to shape the behavior of subjects in real-time as compared to monetary reward. This common social learning mechanism can directly influence the level of localized brain activity using a BCI. Indeed, the social reward led to stronger localized brain activity than the standard feedback. Subjects learned to differentially regulate brain activity depending on the avatar faces. The use of differential stimuli to shape behavior opens new perspectives for developing social feedback paradigms with implicit learning, circumventing explicit cognitive control.

The presence of social reward led to bilateral activation of an anatomically-defined ROI in the corpus striatum (putamen, caudate nucleus, and globus pallidus). These structures belong to a network activated by pleasant and rewarding events (Haber and Knutson, 2010). They are involved in driving incentive-based learning and choosing appropriate responses to stimuli, thereby helping to achieve rewards and avoid punishments, and consequently allow the development of goal-directed behavior (Robbins and Everitt, 1996; Delgado, 2007; Liljeholm and O'Doherty, 2012). Social reward was demonstrated to share comparable neural pathways with monetary reward (Izuma et al., 2008). A number of fMRI and neurophysiology studies confirmed that neural activity in the striatum is modulated by social rewards and by learning in a social context (for a review see Báez-Mendoza and Schultz, 2013; Ruff and Fehr, 2014). Our results are compatible with these studies; moreover we demonstrated that the learning of control over the brain activation improves due to the direct reward.

During the generalization condition, activation in the ACC decreased more in the social feedback group. Although cerebral activation typically increases with higher task load, it is well established that in the course of skill training one can observe the decrease of brain activation (Chein and Schneider, 2005). The effects of training on brain plasticity have been studied in the sensorimotor system, demonstrating a systematic decrease in the motor and somatosensory cortex (Ikegami and Taga, 2008; Kwon et al., 2013; Walz et al., 2014). In trained musicians, gray matter density decreased with expertise in bilateral perirolandic and striatal areas that are related to sensorimotor function, possibly reflecting high automation of motor skills (James et al., 2013). In a similar vein, in a working memory task, the activation in the right inferior frontal gyrus and the right intraparietal sulcus initially increased with improved performance, but decreased when performance consolidated after the prolonged training (Hempel et al., 2004). Moreover, low-performance led to large and load-dependent activation increases in distributed cortical areas when exposed to excessive task requirements, suggesting a recruitment of additional attentional and strategy-related resources by low- as compared to high-performing participants (Jaeggi et al., 2007). In general, the recruitment of a large-scale neural network decreases in the automatic phase, as stimulus-response associations become better and task performance progresses from a consciously controlled manner in the early learning phase to an unconscious form in the late automatic phase (Toni et al., 1998; Müller et al., 2002; Dobbins et al., 2004). Kozasa et al. (2012) compared the performance of trained meditators with non-meditators in a Word-Color-Stroop task, i.e., a cognitive interference task based on a similar principle as the Simon task. Although there were no group differences for the behavioral interference effect, non-meditators activated attention and motor control higher than meditators. The authors suggested that the meditation training improved efficiency via enhanced sustained attention and impulse control. Similarly, in our study, after up to 2 weeks of NF-training, subjects who received social reward could maintain the similar behavioral results in Simon task while engaging less ACC activity than subjects who received standard feedback. The behavioral effects in our study demonstrated an increase of the performance in the Simon task over the training time, reflecting the accompanying decrease in ACC activation due to learning and the corresponding shift from a large network to more specialized regions. In combination with the lack of effects on the behavioral level, we conclude that the social reward led to a reduced neural recruitment to achieve a similar behavioral performance in the Simon task.

Rapid technological advance in fMRI and BCI extends the range of NF applications leading to its increasing popularity. Within the last 2 decades, a number of brain regions were controlled with rt-fMRI NF, including motor areas (deCharms et al., 2004; Yoo et al., 2008), anterior cingulate cortex (Weiskopf et al., 2003; deCharms et al., 2005), supplementary motor and parahippocampal areas (Weiskopf et al., 2004a), anterior insula (Caria et al., 2007; Berman et al., 2013), right inferior frontal gyrus (Rota et al., 2009), amygdala (Zotev et al., 2011; Brühl et al., 2014; Young et al., 2014), nucleus accumbens (Greer et al., 2014), dopaminergic neurons in the substantia nigra/ventral tegmental area complex (Sulzer et al., 2013) or networks of regions, such as individually localized emotion networks (Johnston et al., 2010), the interhemispheric balance between left and right visual cortices (Robineau et al., 2014), or a distributed ensemble of brain regions related to feelings of tenderness/affection (Schoenberg and David, 2014). The first applications of NF in patient groups suggest its potential in the treatment of several disorders, including chronic pain (deCharms et al., 2005), chronic tinnitus (Haller et al., 2010), Parkinson's disease (Subramanian et al., 2011), depression (Linden et al., 2012; Young et al., 2014), obesity (Frank et al., 2012), nicotine addiction (Canterberry et al., 2013; Li et al., 2013), or schizophrenia (Ruiz et al., 2013).

A well-designed feedback system is crucial in order to achieve a successful training of regional brain activation (Sitaram et al., 2008; Sokunbi et al., 2014) and allow its further development into an effective and accurate clinical intervention. Social feedback, offering direct reward for successful regulation, increased the effectiveness of the NF training. We applied a social smile of a changing intensity, which is a very simplified form of social reward. Indeed, more complex social stimulation (including social gestures, prosody, and complex emotional expression) could serve as an even stronger reinforcer and further improve performance. Sokunbi et al. (2014) propose to choose the visual stimuli that relate to the function of the target brain area. In accordance with this view, social feedback could be particularly well fitted to train impaired social interactions in psychiatric patients in implicit learning tasks.

### Limitations

Although we studied a relatively large group of participants for such a complex paradigm, the group size is a limitation. Possibly due to the small group size, we failed to demonstrate stronger ACC regulation during the transfer sessions (regulation without feedback) and behavioral effects on the Simon task in the social feedback group. Despite the high variability of learning success, subgroup analyses with the focus on learners and non-learners are not feasible at this stage. It would be of particular importance to determine the variability between subjects in learning and reward sensitivity during NF and determine predictors for this (Scheinost et al., 2014). In particular, we did not consider the individual learning processes over the three session in 3 days each. Moreover, the test for difference in transfer effects between the social and standard NF might not be optimally selected. Although the test was identical with the learning procedure, it could have a different meaning for both groups. While in the standard feedback the bar in itself presented no rewarding value, it was not the case with the smiling faces. During social NF, subjects received social reward. In the transfer task, they were presented with slightly smiling facial expressions that might have had negative emotional value relative to smiling faces they viewed while regulating successfully. Showing subjects a neutral stimulus while trying to regulate their ACC activation without feedback might improve those results.

The reward system is typically associated with the basal ganglia, but many other brain regions respond to reward as well, including the ACC, the orbital prefrontal cortex, the midbrain dopamine neurons, the dorsal prefrontal cortex, amygdala, hippocampus, thalamus, lateral habenular nucleus, and specific brainstem structures such as the pedunculopontine nucleus and the raphe nucleus (Haber and Knutson, 2010). The exact role of ACC in reward processing is however not fully understood. It has been hypothesized to play a role in sustaining effective choice behavior based on the previous experience (Chudasama et al., 2013) and particularly in anticipation of loss by risky decisions (for a review, see Liu et al., 2011). A recent meta-analysis of brain imaging studies on social decision making in the ultimatum game suggested that the ACC controls and monitors conflicts between emotional and cognitive motivation, in line with its postulated role in general conflict monitoring (Gabay et al., 2014). In this respect, replacing the moving bar with an explicit social reward should not lead to additional ACC involvement, among others, because both tasks require similar involvement to obtain the desired outcome and only the rewarding value of this outcome differs. Although introducing social reward in the NF paradigm improved learning, we cannot rule out a direct impact on reward on the ACC activation, e.g., via increasing the net value of the expected reward (Apps and Ramnani, 2014). Future research may focus on other brain regions to examine if the effect of social reward is universal for all brain structures, or if it specifically facilitates learning in reward-sensitive regions.

Finally, the sequential group allocation based on the order of inclusion does not preclude observer biases. This should be addressed by using random allocation. This in turn, however, may introduce time effects depending on the block size for random allocation (see Tamm and Hilgers, 2014). Another problem in this study design, like in many other feedback trials, is the limited possibility to blind the conditions to the participant as well as the experimenter. In particular for therapeutic trails, this remains a challenge to blind control conditions in fMRI neurofeedback.

## Conclusions

We suggest that social reinforcers can lead to improved learning of self-regulation and improve effects of fMRI-based NF on underlying neural processes such as cognitive interference processing. The advantage of social feedback over standard visual feedback or over monetary rewards is the online provision of a direct external reward that we can experience every day in social interactions. Further research is needed to evaluate if social feedback training has the potential to make the learning process more implicit (deCharms et al., 2005; Sulzer et al., 2013).

## Author contributions

KAM: Development of study paradigm and data analysis. Supervision over data analysis and interpretation. Manuscript revision. EA: Data acquisition, data analysis and interpretation. Manuscript writing. YK: Implementation of toolbox for real-time fMRI and technical support by data acquisition. Revising the manuscript. MD: Contribution to design and data analysis. Revising the manuscript. JC: Contributions to data collection and statistical analysis. Revising the manuscript. TG: Contribution to design and data collection. Revising the manuscript. FZ: Contribution to design. Revising the manuscript. NP: Contribution to development of ACC masks and data analysis. Revising the manuscript. PS: Contribution to data acquisition. Revising the manuscript. SB: Contribution to analysis of behavioral data. Revising the manuscript. MZ: MRI support and technical support by data acquisition. Revising the manuscript. KM: Supervision of and conceptual contributions to study. Data analysis and interpretation. Manuscript revision. All the authors read and approved the final version of the manuscript. All the authors agree to be accountable for all aspects of the work in ensuring that questions related to the accuracy or integrity of any part of the work are appropriately investigated and resolved.

### Conflict of interest statement

The authors declare that the research was conducted in the absence of any commercial or financial relationships that could be construed as a potential conflict of interest.

## Abbreviations

ACC: anterior cingulate cortex
BCI: brain-computer interface
EEG: electroencephalography
EPI: echo planar imaging
FWE: family-wise error (correction)
NF: neurofeedback
ROI: region of interest
rt-fMRI: real-time functional magnetic resonance imaging
SPM: Statistical Parametric Mapping.
