# Supplementary material for: Social reward improves the voluntary control over localized brain activity in fMRI-based neurofeedback training
Source: Front Behav Neurosci. 2015 Jun 3;9:136. doi: 10.3389/fnbeh.2015.00136 (PMC4452886; doi:10.3389/fnbeh.2015.00136)
Supplement: Supplementary file 1 [file DataSheet1.PDF]

Anleitungen für Neurofeedback

- Zuerst: Entspann Dich!
- Es werden zwei Balken erscheinen. Der grüne Balken symbolisiert deine eigene Gehirnaktivität, der rote Balken die zu erreichende Stärke der Aktivität. Versuch deine Aktivität zu steigern, den grünen Balken anzuheben in Richtung des oberen roten Balkens.
- Jede wirksame Strategie braucht mehrere Sekunden um einen Effekt zu zeigen. Deswegen bleib bei jeder Strategie für mindestens 10 sek (oder den ganzen Block) bevor Du wechselst. Wichtig ist, dass Du beobachtest, was passiert
- Strategien können sein:
    - Du erinnerst Dich an ein bestimmtes Ereignis in Deinem Leben – emotionale Erlebnisse funktionieren am Besten
    - Du stellst Dir was vor, z.B. Sport treiben oder ein Instrument spielen
    - Du konzentrierst Dich auf ein bestimmte Wahrnehmung, wie die Temperatur in deinem linken Fuß
- Atme regelmäßig
- Bewege Dich möglichst nicht, aber verkrampfe auch nicht
- Wenn etwas nicht funktioniert, probier was anderes und dann probier es vielleicht noch einmal. Bewerte nicht, sondern beobachte einfach was passiert. Denk daran, dass jede Strategie (wenn nicht geschummelt) mehrere Sekunden braucht um eine Wirkung zu haben.
- Wenn der grüne Balken erscheint, zähle rückwärts (von 100 startend)
- In der Vortestung (und Nachtestung) wird sich auch der grüne Balken nicht bewegen, dann trotzdem eine (erfolgreiche) Strategie anwenden
- Nach jedem Block wirst Du gefragt werden, was für eine Strategie Du genutzt hast und was dann passiert ist.

Anleitungen für Neurofeedback

- Zuerst: Entspann Dich!
- Wenn das blonde/dunkelhaarige Gesicht kommt, versuch ihn lächeln zu lassen:
- Jede wirksame Strategie braucht mehrere Sekunden um einen Effekt zu zeigen. Deswegen bleib bei jeder Strategie für mindestens 10 sek (oder den ganzen Block) bevor Du wechselst. Wichtig ist, dass Du beobachtest, was passiert
- Strategien können sein:
  - Du erinnerst Dich an ein bestimmtes Ereignis in Deinem Leben – emotionale Erlebnisse funktionieren am Besten
  - Du stellst Dir was vor, z.B. Ski zu laufen oder Geige zu spielen
  - Du konzentrierst Dich auf ein bestimmte Wahrnehmung, wie die Temperatur in deinem linken Fuß
- Atme regelmäßig
- Bewege Dich möglichst nicht, aber verkrampfe auch nicht
- Wenn etwas nicht funktioniert, probier was anderes und dann probier es vielleicht noch einmal. Bewerte nicht, sondern beobachte einfach was passiert. Denk daran, dass jede Strategie (wenn nicht geschummelt) mehrere Sekunden braucht um eine Wirkung zu haben.
- Wenn das dunkelhaarige/blonde Gesicht kommt, zähl rückwärts (von 100 startend immer minus 3: 100, 97, 94, …)
- Nach jedem Block wirst Du gefragt werden, was für eine Strategie Du genutzt hast und was dann passiert ist.
- In der Vortestung (und Nachtestung) wird auch das blonde/dunkelhaarige Gesicht nicht lächeln, dann trotzdem eine (erfolgreiche) Strategie anwenden
